# REDD1 deficiency alleviates podocyte PANoptosis and restores autophagy in diabetic kidney disease

**DOI:** 10.1186/s10020-026-01510-8

**Published:** 2026-05-21

**Authors:** Mengyu Liu, Chen Yuan, Yue Li, Shan Song, Lin Mu, Yawei Bian, Xinran Li, Xiaoxue Yu, Guiying Li, Yonghong Shi

**Affiliations:** 1 Department of Pathology, Hebei Medical University, Shijiazhuang, 050017 China; 2 Hebei Key Laboratory of Kidney Disease, Shijiazhuang, 050017 China; 3 Department of Nephrology, Second Hospital of Hebei Medical University, Shijiazhuang, 050051 China; 4 Department of Nephrology, Affiliated Hospital of Hebei University of Engineering, Handan, 056000 China

**Keywords:** Diabetic kidney disease, PANoptosis, Podocyte, Autophagy, REDD1, Stratifin

## Abstract

**Background:**

Podocyte loss and death are pathological hallmarks of diabetic kidney disease (DKD), and PANoptosis (apoptosis, pyroptosis, and necroptosis) in podocytes is crucial to DKD progression. Regulated in development and DNA damage response 1 (REDD1) is a multifaceted regulator involved in metabolism, oxidative stress, autophagy, and cell fate. In this study, we aimed to investigate the effects and underlying mechanisms of REDD1 on podocyte PANoptosis and autophagy in DKD.

**Methods:**

REDD1 knockout (KO) mice were induced to diabetes by intraperitoneal injections of streptozotocin (STZ). We assessed renal function, albuminuria, kidney pathology, and podocyte injury in diabetic mice. In vitro, mouse podocyte cells (MPCs) were transfected with REDD1 shRNA plasmid, stratifin (SFN) expression plasmid, SFN siRNA, and treated with TFEB activator 1 or GSK-872 and cultured in high glucose (HG) medium. Gene and protein expression was assessed by real-time quantitative PCR, western blotting, immunofluorescence, and immunohistochemistry. Apoptosis, cytoskeleton change, mitochondrial morphology and membrane potential were evaluated in podocytes.

**Results:**

REDD1 KO improved renal function and reduced mesangial expansion, podocyte loss, and markers related to PANoptosis in podocytes in diabetic mice. In vitro, REDD1 knockdown suppressed HG-induced PANoptosis, cytoskeletal disorganization, mitochondrial damage, and mitochondrial membrane potential reduction in podocytes. In addition, REDD1 deletion restored autophagy and transcription factor EB (TFEB) expression in diabetic kidneys. Meanwhile, REDD1 knockdown alleviated autophagy dysfunction and promoted TFEB nuclear translocation in podocytes exposed to HG. Moreover, REDD1 KO inhibited podocyte SFN expression in diabetic mice. SFN knockdown or receptor interacting protein kinase 3 (RIPK3) inhibitor GSK-872 alleviated HG-induced PANoptosis and autophagy dysfunction in podocytes. Besides, overexpression of SFN reversed the effect of REDD1 knockdown on PANoptosis and autophagy in HG-treated podocytes.

**Conclusions:**

REDD1 deficiency protects against podocyte injury through inhibiting PANoptosis and restoring autophagy in DKD. REDD1 is a potential therapeutic target to slow the progression of DKD.

## Introduction

Diabetic kidney disease (DKD), a chronic complication of diabetes, has been recognized the most common cause of end-stage renal disease (ESRD) worldwide (Alicic et al. 2017). Approximately 40% of patients with diabetes develop DKD and even progress to ESRD, but effective therapeutic strategies for DKD remain limited (Alicic et al. 2017; Mohandes et al. 2023). Podocytes, terminally differentiated epithelial cells, play an essential role in the formation and maintenance of the glomerular filtration barrier (Torban et al. 2019). Podocyte injury has been recognized as the most important event contributing to the onset of proteinuria and the pathogenesis of DKD (Yamaguchi et al. 2009; Yang et al. 2023; Salemkour et al. 2023). However, effective therapeutic strategies to alleviate podocyte injury in DKD are still lacking. Therefore, it is critical to identify novel therapeutic targets and develop effective therapies for mitigating podocyte injury in DKD.

Programmed cell death (PCD) encompasses multiple precisely regulated forms of cell death, including apoptosis, pyroptosis, necroptosis, ferroptosis, and autophagy, which can be activated by diverse external stimuli and intracellular signals (Qian et al. 2024). Accumulated evidence has shown that various forms of PCD, including apoptosis, autophagy, pyroptosis, necroptosis, and ferroptosis, are associated with podocyte injury in DKD (Liu et al. 2024). Recently, an intertwined cell death modality known as PANoptosis has been identified, which combines key features of pyroptosis, apoptosis, and necroptosis but cannot be explained by any of these alone (Malireddi et al. 2019; Samir et al. 2020; Xiang et al. 2024). Previous studies have shown that pyroptosis, apoptosis, and necroptosis are deeply involved in podocyte damage in DKD (Chen et al. 2025; Zhao et al. 2025; Li et al. 2025). It has been demonstrated that Z-DNA-binding protein 1 (ZBP1)-mediated podocyte PANoptosis is a key driver of renal injury in lupus nephritis (Wang et al. 2025a). Notably, a recent study demonstrated that tumor necrosis factor (TNF)-related apoptosis-inducing ligand (TRAIL)/death receptor 5 (DR5)-mediated podocyte PANoptosis contributed to DKD progression (Lv et al. 2025). Therefore, inhibition of PANoptosis may serve as a potential therapeutic strategy to protect against podocyte injury in DKD.

Autophagy has emerged as a crucial process in podocytes, with growing evidence implicating its dysregulation in the pathogenesis of DKD (Yang et al. 2018). Under stress conditions such as hypoxia, oxidative stress, or endoplasmic reticulum (ER) stress, autophagy is typically activated to promote cell survival (Huber et al. 2012). Previous studies have demonstrated that impaired podocyte autophagy leads to increased cellular damage, whereas restoring autophagy in podocytes alleviates such damage and proteinuria in experimental DKD (Salemkour et al. 2023; Tagawa et al. 2016; Barutta et al. 2023). Notably, crosstalk exists between autophagy and other PCD pathways: autophagy modulates apoptosis by regulating apoptotic factors (Chen et al. 2024), influences pyroptosis via mechanisms involving sequestosome 1 (SQSTM1/p62) and NLRP3 inflammasome regulation (Li et al. 2019), and suppresses necroptosis in intestinal epithelia (Matsuzawa-Ishimoto et al. 2017). Therefore, it is necessary to understand the specific mechanisms underlying podocyte autophagy in DKD and formulate effective therapeutic strategies.

The stress response protein REDD1 (regulated in development and DNA damage response 1, also known as RTP801/Dig2/DDIT4) is a multifaceted regulator involved in metabolism, oxidative stress, autophagy, and cell fate, playing roles in various pathologies including metabolic diseases and cancer (Kim et al. 2023). Our recent study demonstrated that REDD1 expression is increased in the kidneys of diabetic patients and diabetic mice (Mu et al. 2022). REDD1 was involved in apoptosis, epithelial-to-mesenchymal transition (EMT), and oxidative stress in renal tubular epithelial cells exposed to high glucose (Mu et al. 2022). Previous studies have shown that global REDD1 deletion or podocyte-specific REDD1 deletion reduced albuminuria, podocyte loss, and renal injury in diabetic mice (Sunilkumar et al. 2022; Sunilkumar et al. 2025b). In addition, podocyte-specific REDD1 deletion suppressed the activation of the NLRP3 inflammasome and pyroptosis (Sunilkumar et al. 2025a). However, the role and underlying mechanism of REDD1 in diabetes-induced podocyte PANoptosis remain unknown.

In this study, we demonstrate that REDD1 deficiency alleviates podocyte injury and proteinuria in diabetic mice, along with the attenuation of PANoptosis. Mechanistically, we unveil that REDD1 promotes SFN-RIPK3 signaling, which exacerbates podocyte PANoptosis and injury, and impedes the nuclear translocation of TFEB, thereby repressing autophagy. Our findings identify REDD1 as a pivotal upstream regulator of PANoptosis and autophagy in DKD and highlight targeting of REDD1 as a promising therapeutic strategy.

## Materials and methods

### Antibodies and other reagents

Antibodies against β-actin (20536–1-AP), REDD1 (10638–1-AP), p62 (84826–1-RR), B-cell lymphoma 2 interacting coiled-coil protein 1 (Beclin-1, 11306–1-AP), desmin (16520–1-AP), ZBP1 (22803–1-AP), synaptopodin (67339–1-Ig), fibronectin (15613–1-AP), Mixed lineage kinase domain-like pseudokinase (MLKL, 66675–1-Ig), and Histone H3 (17168–1-AP) were purchased from Proteintech Group (Chicago, IL, USA). AIM2 (HY-P87843) was purchased from MedChemExpress (Monmouth Junction, NJ, USA). RIPK1 (ab300617), phosphorylated RIPK1 (p-RIPK1, ab316923), pyrin (ab195975), cleaved caspase-3 (ab214430), collagen IV (ab6586) and 14–3-3σ/SFN (ab14123) antibodies were obtained from Abcam (Cambridge, UK). Phosphorylated RIPK3 (p-RIPK3, 91702) and phosphorylated MLKL (p-MLKL, 37333) antibodies were purchased from Cell Signaling Technology (Danvers, MA, USA). Antibodies against Microtubule-associated protein 1 light chain 3 beta (LC3B, PA1-46286), caspase-8 (PA5-87373) and caspase-1 p20 (PA5-99390) were obtained from Thermo Fisher Scientific (Waltham, MA, USA). Anti-RIPK3 (YM8350) and anti-TFEB (YM8493) antibodies were purchased from Immunoway (Suzhou, China). Anti-nephrin (AF3159) antibody was obtained from R&D Systems (Minneapolis, MN, USA). Anti-WT-1 (ET1610-45) and anti-GSDMD-N (HA721144) antibodies were purchased from Huaan Biotechnology (Hangzhou, China). Streptozotocin (STZ, V900890), D-glucose (G8270), D-mannitol (M4125) and TRITC-phalloidin (FAK100) were purchased from Sigma-Aldrich (St. Louis, MO, USA). TFEB activator 1 (HY-135825), Bafilomycin A1 (BafA1, HY-100558) and GSK-872 (HY-101872) were purchased from MedChemExpress. The blood urea nitrogen (BUN) assay kit (S0574M) was obtained from Beyotime Biotechnology (Shanghai, China). Creatinine (Cr) assay kit (BC4915) was purchased from Solarbio Science & Technology (Beijing, China). REDD1 shRNA plasmids, SFN plasmid and SFN siRNA were obtained from GenePharma (Shanghai, China).

### Animals

Heterozygous REDD1 knockout (REDD1^+/-^) mice were purchased from Gempharmatech Co., Ltd (Nanjing, China). Homozygous REDD1 knockout (REDD1^−/−^) male mice were used for experiments. The genotyping of the REDD1 KO mice was confirmed through PCR from genomic DNA isolated from toes with the following primers: Forward 5'-CTTGGCTGCCCTGAAATTGC-3'; Reverse 5'-TTTGTGGTTGGTGGGAGATGTG-3'. Wild-type (WT) littermates were used as control. Mice were housed at the Public Service Platform for Laboratory Animals of Hebei Medical University at an ambient temperature of 22℃ with a 12:12 h light: dark cycle and free access to food and water. Diabetes was induced by intraperitoneal injections of STZ (50 mg/kg daily for 5 days) dissolved in 0.1 M sodium citrate buffer (pH 4.5). At 3 days after injection, blood glucose > 16.7 mmol/l was defined as successful modeling. After 16 weeks of feeding, 24 h urine was collected. Mice were sacrificed, and kidneys were harvested, weighed, dissected, and snap-frozen or paraffin processed for further analysis. All animal experiments were performed in accordance with the National Institutes of Health Guidelines for the Care and Use of Laboratory Animals and approved by the Experimental Animal Welfare Ethics Committee of Hebei Medical University (IACUC-Hebmu-2024065).

### Histology and immunohistochemistry

Kidneys were fixed overnight in 4% paraformaldehyde, embedded in paraffin, and made into 4 μm sections. Sections were stained using a Periodic Acid-Schiff (PAS) staining kit (G1281, Solarbio Science & Technology) according to the manufacturer's protocol. Quantification of mesangial area and glomerular volume was performed as described (Zuo et al. 2024; Flyvbjerg et al. 2002). For immunohistochemistry of WT-1, sections were incubated with anti-WT-1 antibody (1:100) at 4 °C overnight, followed by appropriate biotinylated secondary antibody. Signals were developed using 3,3’-diaminobenzidine (DAB), and images were captured with an Olympus microscope (Japan). Podocyte number was counted as the number of WT-1^+^ nuclei per glomerular section on at least 30 glomeruli per mouse.

### Cell culture

Conditionally immortalized mouse podocyte cells (MPCs) purchased from the Cell Culture Center (PUMC, CAMS) were cultured as previously described (Wu et al. 2021). Undifferentiated MPCs were cultured in DMEM-F12 medium containing 10% FBS, 10–50 U/ml mouse recombinant γ-IFN, 100 U/ml penicillin and 100 μg/ml streptomycin at 33 °C in a 5% CO_2_ incubator. At 37 °C, MPCs were cultured with DMEM-F12 medium containing 100 U/ml penicillin and 100 μg/ml streptomycin without adding mouse recombinant γ-IFN for 10–15 days to induce cell differentiation and maturation. Cells were cultured in serum-free DMEM-F12 medium for 6 h before experiments. Transfections of podocytes with REDD1 shRNA plasmid (pGPU6-GFP-Neo-shREDD1), SFN plasmid (pcDNA3.1-SFN), SFN siRNA and their negative controls were conducted via Lipofectamine 3000 (L3000015, Thermo Fisher Scientific). The podocytes were treated with TFEB activator 1 (1 μM), BafA1 (100 nM) and GSK-872 (3 μM) respectively under normal glucose (NG, 5.6 mM) or high glucose (HG, 30 mM) conditions for 48 h. NG plus mannitol (M, 24.4 mM) was used as an osmotic control.

### Western blot

Total protein was extracted from renal cortex and MPCs using RIPA lysis buffer. Nuclear and cytoplasmic proteins were extracted using a Nucleoprotein Extraction Kit (R0050, Solarbio Science & Technology). Protein concentrations were determined via BCA assay kit (KGB2101-500, KeyGen Biotech, Nanjing, China). Protein loading was adjusted to ensure that all bands fell within the linear range of detection, as determined by preliminary experiments with serial dilutions. Equal amounts of protein were separated by SDS-PAGE and transferred to PVDF membranes (Millipore, Billerica, MA, USA). Membranes were incubated with antibodies against REDD1, collagen IV, nephrin, desmin, synaptopodin, fibronectin, SFN, TFEB, LC3B, p62, Beclin-1, AIM2, Pyrin, ZBP1, RIPK1, p-RIPK1, RIPK3, MLKL, p-RIPK3, p-MLKL, Histone H3, GSDMD-N, caspase-1 p20, cleaved caspase-3, caspase-8, and β-actin overnight at 4 °C. After incubation with HRP-conjugated secondary antibodies, blots were developed using an enhanced chemiluminescence substrate (BL520B, Biosharp Life Sciences, Beijing, China) and visualized using an Amersham Imager 600. Band intensities were quantified with ImageJ software.

### Real-time quantitative PCR (RT-qPCR)

Total RNA was extracted from kidney tissues and MPCs using TRIzol reagent (DP424, TIANGEN Biotech, Beijing, China) and cDNA was prepared using a commercial kit (MR05101, Monad Biotech, Shanghai, China) according to the instructions. The primers used were: fibronectin, sense 5'-GAACAGTGGCAGAAAGAATA-3', antisense 5'-CAGGTCTACGGCAGTTGT-3'; collagen IV, sense 5′-TCGGACCCACTGGTGATAAA −3′, antisense 5′-AAGCCCATTCCTCCAACTGA-3′; SFN, sense 5′-GGAGAGAGCCAGTCTGATCC-3′, antisense 5′-GCTGCCATGTCTTCATACCG-3′. 18S rRNA, sense 5′-ACACGGACAGGATTGACAGA-3′, and antisense 5′-GGACATCTAAGGGCATCACAG-3′. RT-qPCR was performed using SYBR Premix Ex TaqTM II according to the manufacturer's instructions. Reactions were performed in triplicate on an Agilent Mx3000P qPCR system (Agilent, CA, USA), and relative gene expression was calculated using the 2^–ΔΔCt^ method.

### Immunofluorescence

Kidney Sects. (4 µm) were prepared as above. After blocking with goat serum for 30 min at 37 °C, the sections were incubated overnight at 4 °C with primary antibodies against nephrin (1:100), REDD1 (1:100), SFN (1:50), synaptopodin (1:100), desmin (1:100), collagen IV (1:100), fibronectin (1:100), GSDMD-N (1:100), caspase-1 p20 (1:100), cleaved caspase-3 (1:100), p62 (1:200), LC3B (1:100), TFEB (1:100), p-RIPK3 (1:100), or p-MLKL (1:50). After washing, sections were incubated with FITC- or TRITC- conjugated secondary antibodies. Nuclei were counterstained with DAPI. Images were acquired using a laser confocal microscope (SP8, Leica, Germany) and analyzed with ImageJ software (NIH).

MPCs were washed with PBS and fixed with 4% paraformaldehyde for 30 min at room temperature, and treated with 0.3% Triton X-100 about 10 min at 37 °C, then incubated with goat serum about 30 min at 37 °C. Cells were incubated with antibodies against SFN (1:200), TFEB (1:100), p62 (1:100), LC3B (1:200), p-RIPK3 (1:100), cleaved caspase-3 (1:100), or GSDMD-N (1:100) overnight at 4 °C. FITC- or TRITC- conjugated secondary antibodies were applied for 2 h at 37 °C. Images were captured using a laser confocal microscope (SP8, Leica, Germany).

### Flow cytometry

Measurement of cell apoptosis by flow cytometry analysis was operated using an Annexin V-FITC/PI apoptosis detection kit (HY-K1073, MedChemExpress) according to the manufacturer's instructions. Briefly, cells were stained with 5 µl of Annexin V-FITC for 15 min at room temperature in the dark, followed by the addition of 10 µl of PI for 15 min. The cells were collected and analyzed using a flow cytometer (BD FACS Calibur; BD Biosciences), and the data were analyzed using the FlowJo 8.1 software.

### TUNEL assay

Podocyte apoptosis was measured using Cell Meter TUNEL Apoptosis Assay Kit (T2130, Solarbio Science & Technology) according to manufacturer’s instructions. Images were captured using an Olympus BX51 microscope. TUNEL-positive apoptotic cells were counted in six different fields for each sample and then averaged.

### Mitochondrial morphology and membrane potential

MPCs were stained with MitoTracker™ Red CMXRos (200 nM; M46752, Thermo Fisher Scientific) for 30 min at 37 °C. After fixation, images were acquired using a Leica SP8 confocal microscope. Mitochondrial length was quantified using ImageJ software as previously described (Xiao et al. 2022).

Mitochondrial membrane potential (MMP) was assessed using the JC-1 kit (C2006, Beyotime Biotechnology) according to the manufacturer’s instructions. MPCs were incubated with JC-1 working solution for 30 min at 37 °C and imaged immediately using confocal microscopy. The mean fluorescence intensity for both red (aggregates) and green (monomers) channels within the regions of interest (ROIs) was measured. Quantitative analysis of the fluorescence intensity was performed using ImageJ software (NIH). The ΔΨm for each field was expressed as the ratio of mean red fluorescence intensity to mean green fluorescence intensity (Red/Green ratio).

### Staining of the F-actin cytoskeleton

MPCs were fixed with 4% paraformaldehyde at 4 °C for 30 min and stained with 2.5 μg/ml TRITC-phalloidin (FAK100, Sigma-Aldrich) overnight at room temperature. All microscopy images were recorded using a confocal microscope. The cortical F-actin score (CFS) was assessed to quantify the degree of cytoskeletal reorganization.

### Tandem mRFP-GFP-LC3 fluorescence microscopy

The autophagy flux was observed by tandem mRFP-GFP-LC3 fluorescence microscopy. After 48 h transfection of MPCs with adenovirus expressing mRFP-GFP-LC3 (Hanbio, Shanghai, China), images were observed and collected by confocal laser scanning microscope.

### Co-immunoprecipitation (Co-IP assay)

MPCs were lysed in RIPA buffer on ice for 30 min and centrifuged at 13,523 × g for 30 min at 4 °C. Diverse groups were incubated with different primary antibodies overnight at 4 °C with gentle shaking. Protein A-agarose beads (sc-2003, Santa Cruz Biotechnology, Dallas, TX, USA) were then added and incubation continued for an additional 4 h at 4 °C. After washing three times, the beads were boiled with 1 × buffer for 7 min, and then subjected to immunoblot analysis.

### Cycloheximide (CHX) chase assay

MPCs with stable REDD1 knockdown and corresponding control cells were treated with CHX (MedChemExpress, HY-12320, 30 µg/mL) under HG conditions. Cells were harvested at indicated time points (0, 2, 4, and 8 h) after CHX treatment. Cell lysates were prepared using RIPA lysis buffer and subjected to western blot analysis to determine the half-life of SFN protein.

### Statistical analysis

All quantitative data are expressed as the mean ± SEM. Statistical analyses were performed using GraphPad Prism software (version 9.0, GraphPad Software, USA). For comparisons between two groups, an unpaired two-tailed Student t test was applied. For comparisons among three or more groups, one-way analysis of variance (ANOVA) was used, followed by Tukey’s post hoc test for multiple comparisons. A *p*-value < 0.05 was considered statistically significant.

## Results

### REDD1 deletion prevents renal injury in diabetic mice

**Fig. 1:**
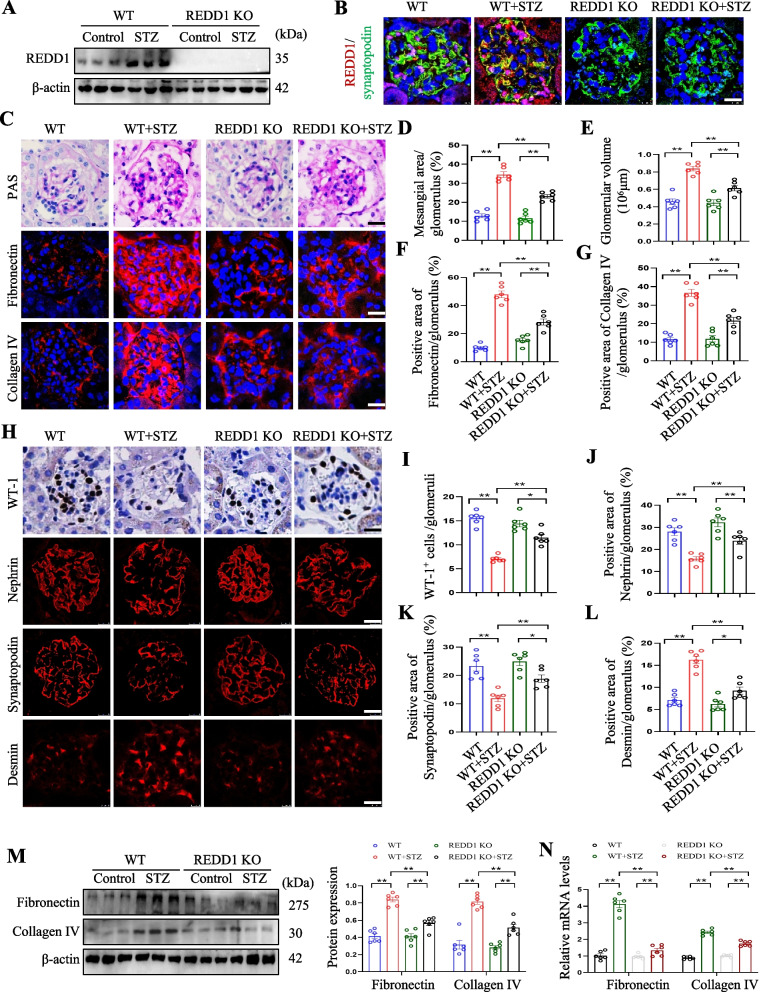
REDD1 knockout alleviates kidney injury in diabetic mice*. ***A** Western blot analysis of REDD1 expression in the renal tissues of mice. **B** Representative immunofluorescence confocal images for REDD1, podocyte marker synaptopodin, and DAPI in kidney sections (scale bar, 20 μm). **C** Representative images of PAS-stained kidney sections and immunofluorescence staining for fibronectin and collagen IV (scale bars, 20 μm). **D**, **E** Quantification of the mesangial area and volume of glomeruli. **F**,** G** Quantitative analysis of fibronectin and collagen IV expression in glomeruli.** H** Representative images of immunohistochemical staining for the podocyte marker WT-1 (scale bar, 20 μm) and immunofluorescence staining for synaptopodin, nephrin, and desmin in glomeruli (scale bar, 20 μm). **I** Quantification of podocyte number based on WT-1^+^ nuclei. **J**-**L** Quantifications of expressions of nephrin, synaptopodin and desmin in glomeruli. **M** Representative western blots and the quantification of fibronectin and collagen IV protein levels in the renal cortex. **N** Renal mRNA levels of fibronectin and collagen IV were analyzed by RT-qPCR. Each bar represents the mean ± SEM for groups of six mice. *P < 0.05, **P < 0.01

To evaluate the role of REDD1 in diabetic kidney injury, we generated REDD1 KO mice. The expression of REDD1 was significantly increased in diabetic kidneys, and the knockout efficiency of REDD1 was confirmed by western blot (Fig. 1A). Meanwhile, immunofluorescence double staining confirmed that REDD1 expression was significantly increased in podocytes of diabetic mice, and REDD1 expression in podocytes was not detected in REDD1 KO mice (Fig. 1B). As shown in Table 1, blood glucose levels were significantly elevated in all STZ-induced diabetic mice compared to non-diabetic controls. Blood glucose level was not affected by REDD1 KO. Diabetic WT mice exhibited significant increases in both the kidney weight/body weight ratio and 24-h urine albumin excretion (UAE), which were attenuated by REDD1 KO. Additionally, REDD1 KO reduced urinary albumin-to-creatinine ratio (UACR) and levels of serum creatinine (Scr) and BUN in diabetic mice, indicating that REDD1 KO improved renal function in diabetic mice.

**Table 1:** Animal characteristics

Parameters	WT	WT + STZ	REDD1 KO	REDD1 KO + STZ
Blood glucose (mmol/L)	7.92 ± 1.00	28.63 ± 1.31**	7.53 ± 0.72	27.60 ± 1.72
Body weight (g)	26.63 ± 1.33	22.62 ± 1.77**	28.34 ± 1.28	25.83 ± 0.99^##^
kidney weight (mg)	310 ± 32.86	365 ± 28.81**	330 ± 25.30	340 ± 17.89
Kidney/Body weight (mg/g)	11.63 ± 0.85	16.16 ± 1.05**	11.64 ± 0.70	13.17 ± 0.61^##^
UAE (mg/24 h)	14.53 ± 0.61	64.77 ± 4.76**	14.13 ± 0.95	46.79 ± 2.66^##^
Scr (μmol/L)	14.86 ± 0.76	35.98 ± 2.97**	13.54 ± 1.37	23.68 ± 3.74^##^
UACR (mg/g)	21.73 ± 2.13	51.02 ± 3.81**	21.28 ± 3.34	38.43 ± 2.85^##^
BUN (mmol/L)	3.53 ± 0.80	9.01 ± 1.66**	3.45 ± 0.93	5.44 ± 1.51^##^

*WT* Wild-type, *STZ* Streptozotocin, *REDD1 KO* REDD1 knockout, *UAE* Urine albumin excretion, *Scr* Serum creatinine, *UACR* Urine albumin/creatinine ratio, *BUN* Blood urea nitrogen. Data are presented as mean ± SEM (*n* = 6)

^**^P < 0.01 vs. WT group

^##^*P* < 0.01 vs. WT + STZ group

After 16 weeks of diabetes, the diabetic WT mice exhibited significant mesangial expansion and glomerular hypertrophy compared to nondiabetic mice, which were retarded by REDD1 KO (Fig. 1C-E). Immunofluorescence staining demonstrated that REDD1 KO suppressed glomerular fibronectin and collagen IV expression levels in diabetic mice (Fig. 1C, F, G). A pronounced loss of podocytes, indicated by a reduction in WT-1^+^ cells in glomeruli, was observed in diabetic mice, which could be restored by REDD1 KO (Fig. 1H, I). In addition, the podocyte injury was further supported by decreased expression of nephrin and synaptopodin, and increased expression of desmin in diabetic mice, and these alterations were reversed by REDD1 KO (Fig. 1H, J-L). Moreover, we found that REDD1 KO significantly suppressed protein and mRNA levels of fibronectin and collagen IV in kidney tissues of diabetic mice (Fig. 1M, N).

### REDD1 ablation ameliorates podocyte PANoptosis in diabetic mice

**Fig. 2:**
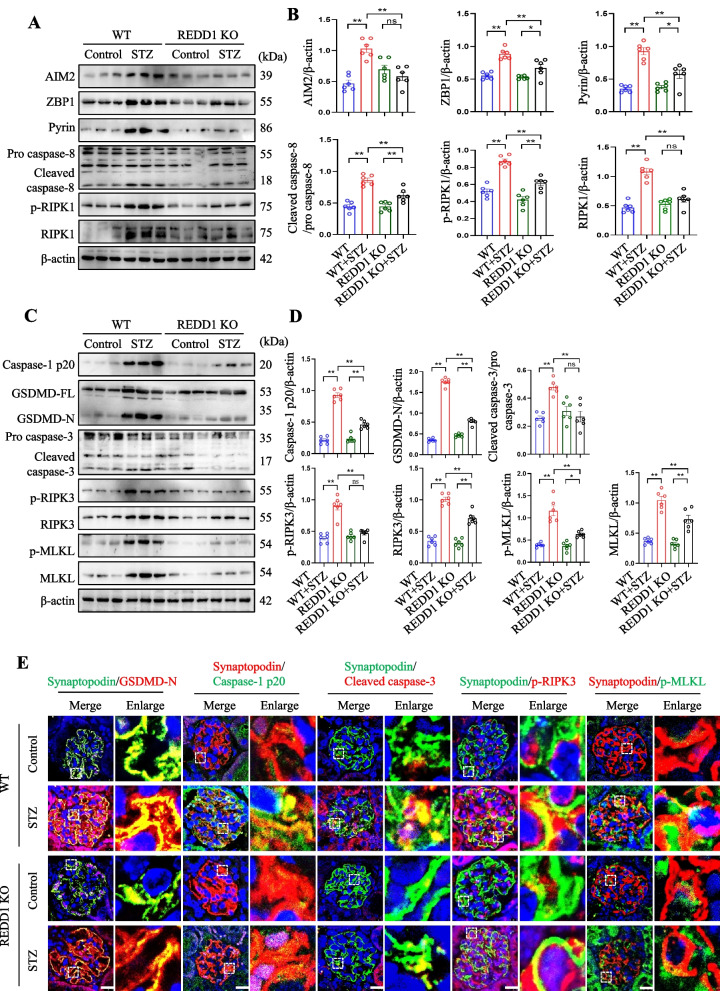
REDD1 ablation inhibits podocyte PANoptosis in diabetic mice. **A**, **B** Representative western blot images and quantitative analysis of PANoptosome components (AIM2, ZBP1, Pyrin, caspase-8, RIPK1 and p-RIPK1) in renal tissues from control and diabetic mice with or without REDD1 deletion. **C**, **D** Protein expression and quantitative analysis of key molecular markers governing pyroptosis (caspase-1 p20 and GSDMD-N), apoptosis (cleaved caspase-3) and necroptosis (p-RIPK3, RIPK3, p-MLKL, and MLKL) in mouse kidney tissues. **E** Immunofluorescence double staining of PANoptosis-related markers in renal podocytes of each group (scale bar, 20 μm). Each bar represents the mean ± SEM for groups of six mice. ns: no significance. *P < 0.05, **P < 0.01

Necroptosis, pyroptosis, and apoptosis play important roles in podocyte injury and are involved in the pathogenesis of DKD (Yang et al. 2023). PANoptosis is executed by a multiprotein complex activated by the DNA sensors AIM2, ZBP1, and Pyrin. These sensors assemble into a flexible scaffold that recruit caspase-1, caspase-3, caspase-8, RIPK1, and RIPK3, leading to the simultaneous execution of pyroptosis, apoptosis, and necroptosis (Bi et al. 2022). Our results demonstrated that PANoptosis in the kidneys of diabetic mice is characterized by elevated levels of key PANoptosome components, including AIM2, ZBP1, Pyrin, caspase-8, and RIPK1, as well as increased phosphorylation of RIPK1. Notably, REDD1 deficiency reduced the expression of these proteins in diabetic kidneys (Fig. 2A, B). In addition, the expression of caspase-1 p20 and GSDMD-N (markers of pyroptosis), cleaved caspase-3 (markers of apoptosis), as well as RIPK3, MLKL, p-RIPK3 and p-MLKL (markers of necroptosis) was markedly upregulated in diabetic kidneys, which was suppressed by REDD1 ablation (Fig. 2C, D). In addition, we evaluated the effect of REDD1 KO on podocyte PANoptosis in diabetic mice. Immunofluorescence double staining revealed that REDD1 KO significantly reduced the elevated expression of GSDMD-N, caspase-1 p20, cleaved caspase-3, p-RIPK3 and p-MLKL in podocytes of diabetic mice (Fig. 2E).

### REDD1 contributes to PANoptosis in podocytes exposed to HG

**Fig. 3:**
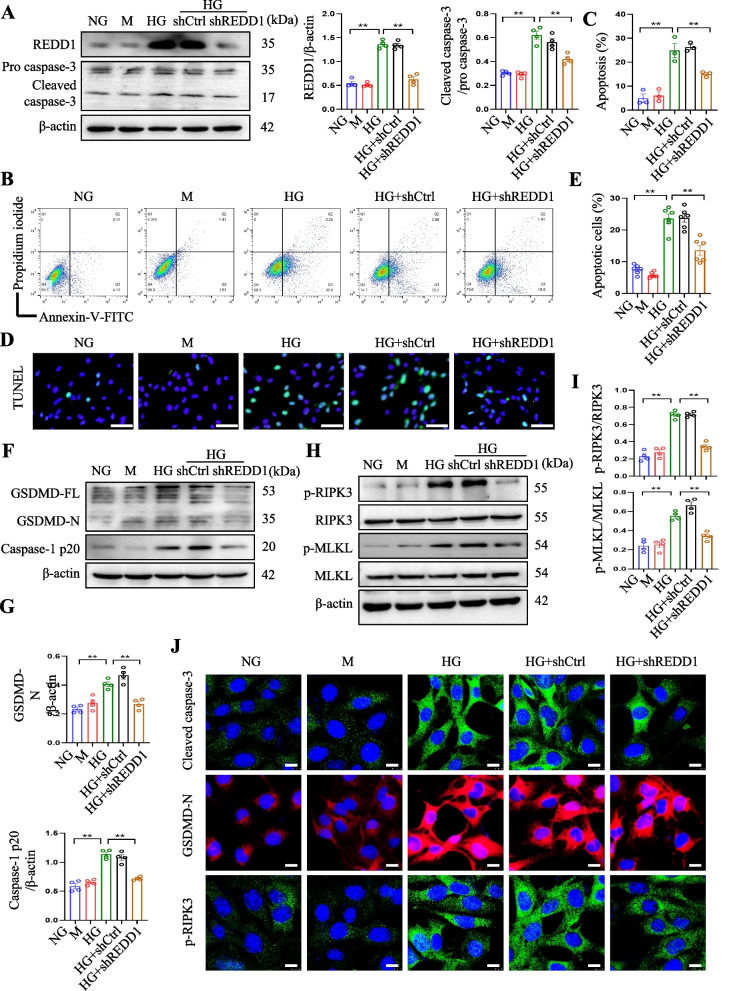
REDD1 knockdown suppresses HG-induced PANoptosis in podocytes. **A** Representative western blot and the quantitative analysis of REDD1 and cleaved caspase-3 levels in MPCs transfected with control (shCtrl) or REDD1-targeting shRNA (shREDD1) and exposed to NG or HG conditions (*n* = 4). **B**, **C** Representative flow cytometry plots and quantification of apoptotic MPCs (Annexin V-positive) under the indicated conditions (*n* = 3). **D**,** E** Representative TUNEL staining images (scale bar, 50 μm) and quantitative analysis of TUNEL-positive MPCs (*n* = 6). **F**, **G** Representative western blot and the quantitative analysis of pyroptosis-related proteins (GSDMD-N and caspase-1 p20) in MPCs (*n* = 4). **H**,** I** Representative western blot and the quantitative analysis of necroptosis-related proteins (total and phosphorylated RIPK3 and MLKL) in MPCs (*n* = 4). **J** Representative immunofluorescence images for cleaved caspase-3, GSDMD-N, and p-RIPK3 in MPCs (scale bar, 7.5 μm). NG: 5.6 mM D-glucose; M: NG + mannitol (24.4 mM); HG: 30 mM D-glucose; shCtrl: shRNA control plasmid; shREDD1: REDD1 shRNA plasmid. Data are presented as mean ± SEM. **P < 0.01

**Fig. 4:**
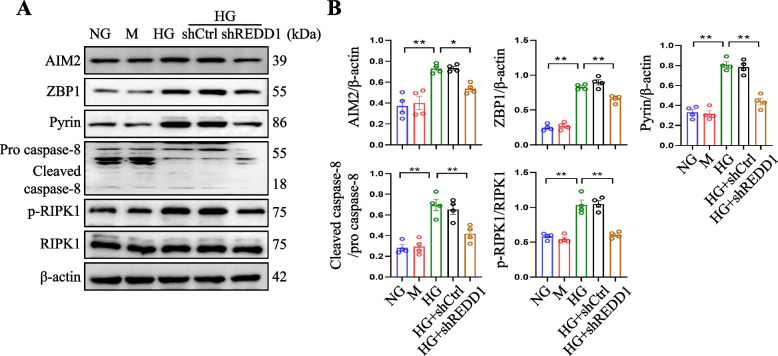
REDD1 knockdown attenuates HG-induced PANoptosome component upregulation in podocytes. **A**,** B** Western blot analysis and quantitative analysis of PANoptosome components, including AIM2, ZBP1, Pyrin, caspase-8, RIPK1, and p-RIPK1. NG: 5.6 mM D-glucose; M: NG + mannitol (24.4 mM); HG: 30 mM D-glucose; shCtrl: shRNA control plasmid; shREDD1: REDD1 shRNA plasmid. Data are presented as mean ± SEM (*n* = 4). *P < 0.05, **P < 0.01

A recent study has demonstrated that podocyte PANoptosis (apoptosis, pyroptosis, and necroptosis) is involved in podocyte loss in DKD (Lv et al. 2025). Therefore, we evaluated the effect of REDD1 interference on PANoptosis in MPCs under HG conditions. HG significantly upregulated REDD1 expression in MPCs, and this change was retarded by REDD1 shRNA transfection (Fig. 3A). The increased expression of cleaved caspase-3 induced by HG was suppressed by REDD1 silencing (Fig. 3A). Meanwhile, flow cytometry and TUNEL staining showed that the proportion of apoptotic cells increased in HG-treated MPCs, and this alteration was reversed by REDD1 knockdown (Fig. 3B-E). In addition, the expression of pyroptosis markers (caspase-1 p20 and GSDMD-N) and necroptosis markers (p-RIPK3 and p-MLKL) was increased in HG-treated MPCs, which was suppressed by REDD1 knockdown (Fig. 3F-I). Furthermore, immunofluorescence staining confirmed that HG-induced expression of cleaved caspase-3, GSDMD-N, and p-RIPK3 was reduced by REDD1 knockdown (Fig. 3J). Notably, the levels of PANoptosome components, including AIM2, ZBP1, Pyrin, caspase-8, and p-RIPK1, were increased in HG treated MPCs, and these alterations were suppressed by REDD1 knockdown (Fig. 4A, B). Taken together, these data suggest that REDD1 knockdown prevents the activation of PANoptosis pathway in podocytes under HG conditions.

### REDD1 knockdown alleviates HG-induced podocyte injury, cytoskeletal disruption, and mitochondrial damage

**Fig. 5:**
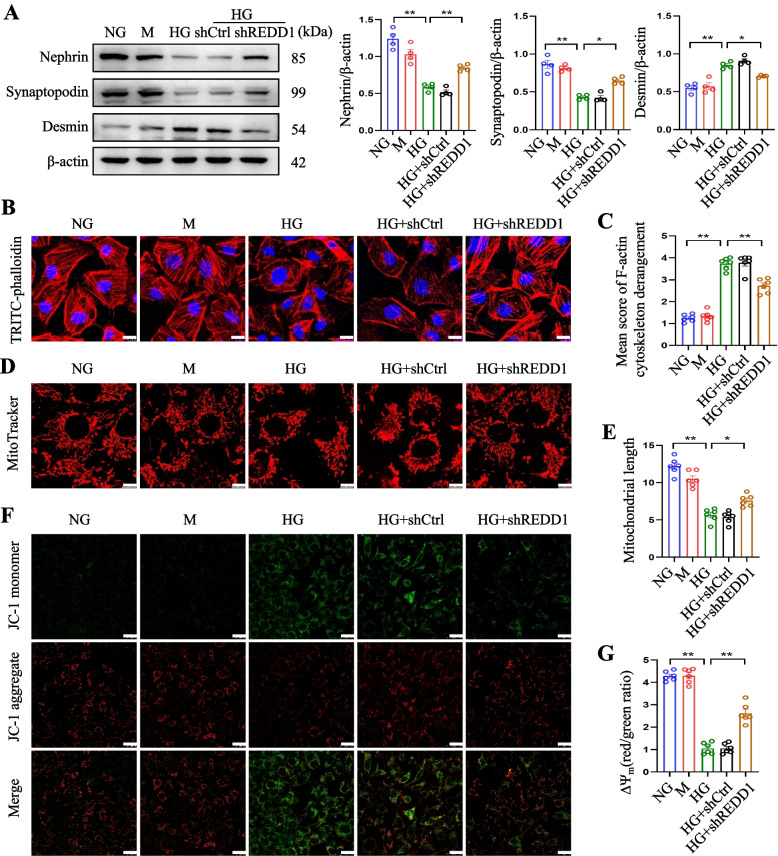
Effect of REDD1 knockdown on HG-induced podocyte injury, cytoskeleton, and mitochondria*. ***A** Representative western blot images and quantification of nephrin, synaptopodin, and desmin in MPCs (*n* = 4). **B** Representative TRITC-phalloidin staining in podocytes (scale bar, 10 μm). **C** Quantitative analysis of actin cytoskeleton derangement (*n* = 6). **D**, **E** Representative images and quantification of mean mitochondrial length in MPCs stained with MitoTracker Red (scale bar, 10 μm, *n* = 6). **F**,** G** Representative images and quantitative analysis of MMP in MPCs stained with JC-1 dye (scale bar, 25 μm, *n* = 6). The red/green fluorescence intensity ratio indicates MMP. NG: 5.6 mM D-glucose; M: NG + mannitol (24.4 mM); HG: 30 mM D-glucose; shCtrl: shRNA control plasmid; shREDD1: REDD1 shRNA plasmid. Data are presented as mean ± SEM. *P < 0.05, **P < 0.01

Next, we investigated the effect of REDD1 interference on HG-induced podocyte injury in vitro. Our results indicated that HG-induced podocyte injury, characterized by decreased nephrin and synaptopodin expression and increased desmin expression, was reversed by REDD1 knockdown (Fig. 5A). We next evaluated the effect of REDD1 silencing on cytoskeleton change using F-actin staining in MPCs under HG conditions. Following 48 h of HG exposure, MPCs exhibited disorganized stress fibers, characterized by peripheral actin bundles and scattered fragments, and this cytoskeletal disarray was restored by REDD1 shRNA (Fig. 5B, C). In addition, we examined mitochondrial morphology using MitoTracker Red staining. We found that HG-induced reduction in mean mitochondrial length was reversed by REDD1 knockdown (Fig. 5D, E). Moreover, the loss of MMP caused by HG was also restored by REDD1 knockdown in MPCs (Fig. 5F, G).

### REDD1 deficiency ameliorates podocyte autophagy disorder

**Fig. 6:**
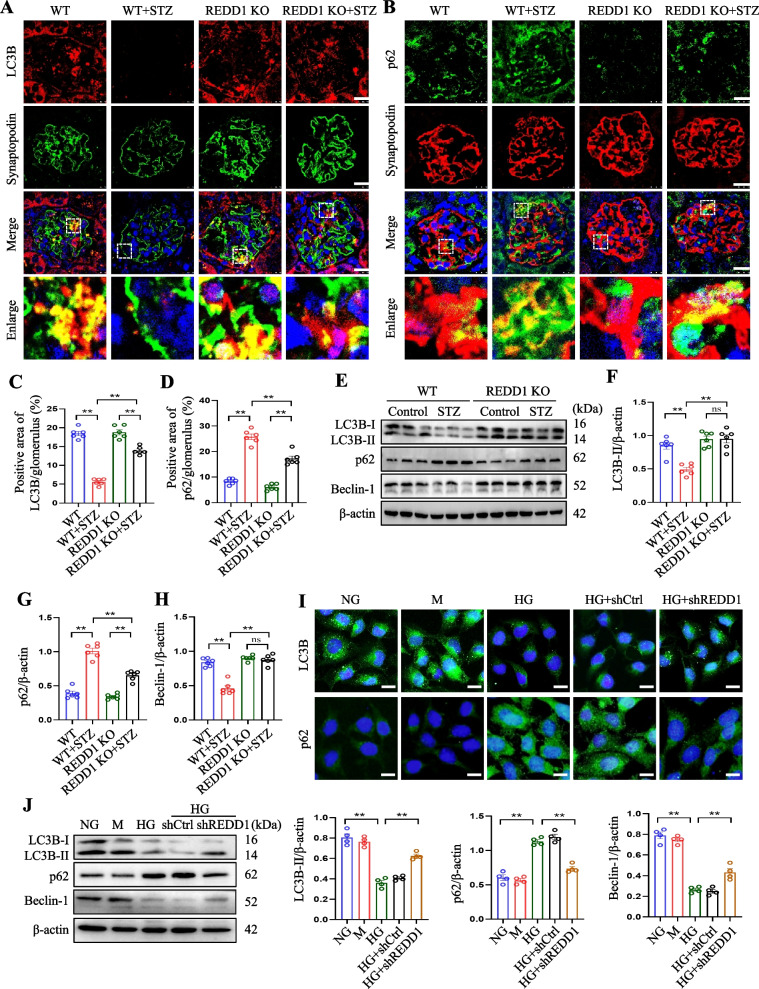
REDD1 deficiency restores autophagy in podocytes under diabetic conditions*. ***A** Representative immunofluorescence confocal images for LC3B, synaptopodin, and DAPI in kidney sections (scale bar, 20 μm). **B** Representative immunofluorescence confocal images for p62, synaptopodin, and DAPI in kidney sections (scale bar, 20 μm). **C**, **D** Quantification of the percentage area of LC3B puncta and p62 expression in glomeruli. (*n* = 6). **E**–**H** Representative western blot images and quantification of LC3B, p62, and Beclin-1 in renal cortex (*n* = 6). **I** Representative immunofluorescence images for LC3B and p62 in MPCs (scale bar, 10 μm). **J** Representative western blot images and quantification of LC3B, p62, and Beclin-1 in MPCs (*n* = 4). NG: 5.6 mM D-glucose; M: NG + mannitol (24.4 mM); HG: 30 mM D-glucose; shCtrl: shRNA control plasmid; shREDD1: REDD1 shRNA plasmid. Data are presented as mean ± SEM. ns: no significance. **P < 0.01

**Fig. 7:**
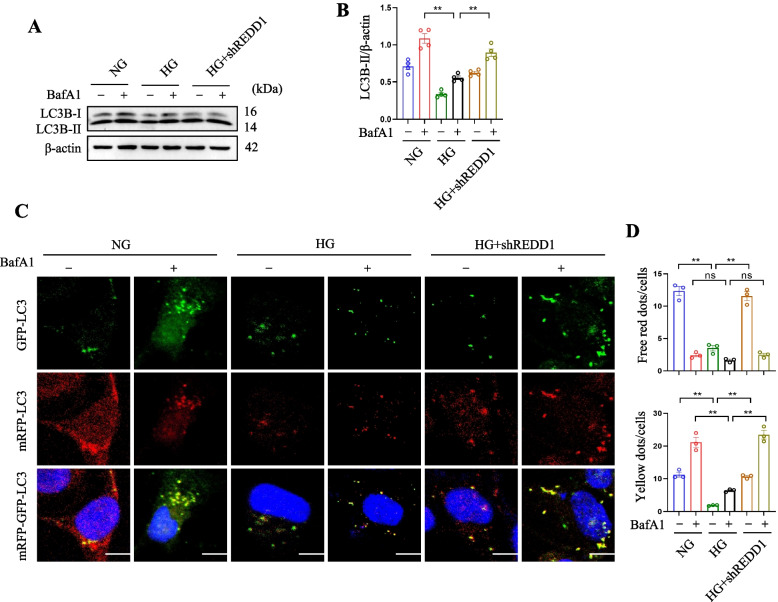
REDD1 knockdown restores HG-impaired autophagic flux in podocytes. **A**, **B** Western blot analysis and quantitative analysis of LC3B expression in MPCs with or without BafA1 intervention under NG and HG conditions (*n* = 4). **C**, **D** Representative images of mRFP-GFP-LC3 puncta and quantitative analysis of yellow (autophagosomes) and red (autolysosomes) puncta in each group (scale bar, 7.5 μm). NG: 5.6 mM D-glucose; HG: 30 mM D-glucose; shREDD1: REDD1 shRNA plasmid. Data are presented as mean ± SEM. ns: no significance. **P < 0.01

Impaired podocyte autophagy has been closely associated with podocyte injury in DKD (Tagawa et al. 2016). A previous study has demonstrated that REDD1-mediated autophagy was involved in senescence in retinal pigment epithelium (Chen et al. 2022). However, it remains unclear whether REDD1 contributes to podocyte autophagy dysregulation in DKD. Immunofluorescence double staining revealed a reduction in punctate LC3B staining along with a significant increase in p62 in podocytes of diabetic mice, and these changes were markedly reversed by REDD1 deletion (Fig. 6A, B). Correspondingly, the reduction in LC3B expression along with the increase in p62 within glomeruli of diabetic mice was significantly reversed by REDD1 KO (Fig. 6A-D). Consistent with these findings, western blot analysis showed that the decreased levels of LC3B-II and Beclin-1 and increased expression of p62 in diabetic kidney tissues were reversed by REDD1 KO, indicating that REDD1 mediates impaired autophagic flux in diabetic kidneys (Fig. 6E-H). In addition, immunofluorescence revealed a significant reduction in LC3B expression accompanied by a marked accumulation of p62 in MPCs exposed to HG, which were significantly reversed by REDD1 interference (Fig. 6I). Similarly, the decreased levels of LC3B-II and Beclin-1 and increased expression of p62 induced by HG were reversed by REDD1 knockdown in MPCs (Fig. 6J). To exclude the potential influence of altered autophagic turnover on the observed decrease in autophagosome number, we blocked autophagic degradation using the lysosomal inhibitor BafA1. We found that BafA1-induced LC3B-II accumulation was reduced by HG stimulation, whereas REDD1 knockdown restored this change (Fig. 7A, B). To assess autophagic flux and distinguish impaired formation from defective degradation, we performed mRFP-GFP-LC3 assay with or without BafA1. In the absence of BafA1, HG decreased both autophagosome (yellow) and autolysosome (red) numbers, and REDD1 knockdown restored these deficits (Fig. 7C, D). In the presence of BafA1, HG-treated MPCs exhibited strongly reduced autophagosome (yellow) accumulation compared to NG controls, whereas this alteration was restored by REDD1 knockdown, confirming that REDD1 deficiency reverses HG-induced impairment of autophagosome formation (Fig. 7C, D). Collectively, these results demonstrate that HG suppresses autophagic flux by inhibiting autophagosome biogenesis, and that REDD1 deficiency rescues autophagic flux in podocytes under HG conditions.

### REDD1 deficiency promotes TFEB expression and nuclear translocation in podocytes under diabetic conditions

**Fig. 8:**
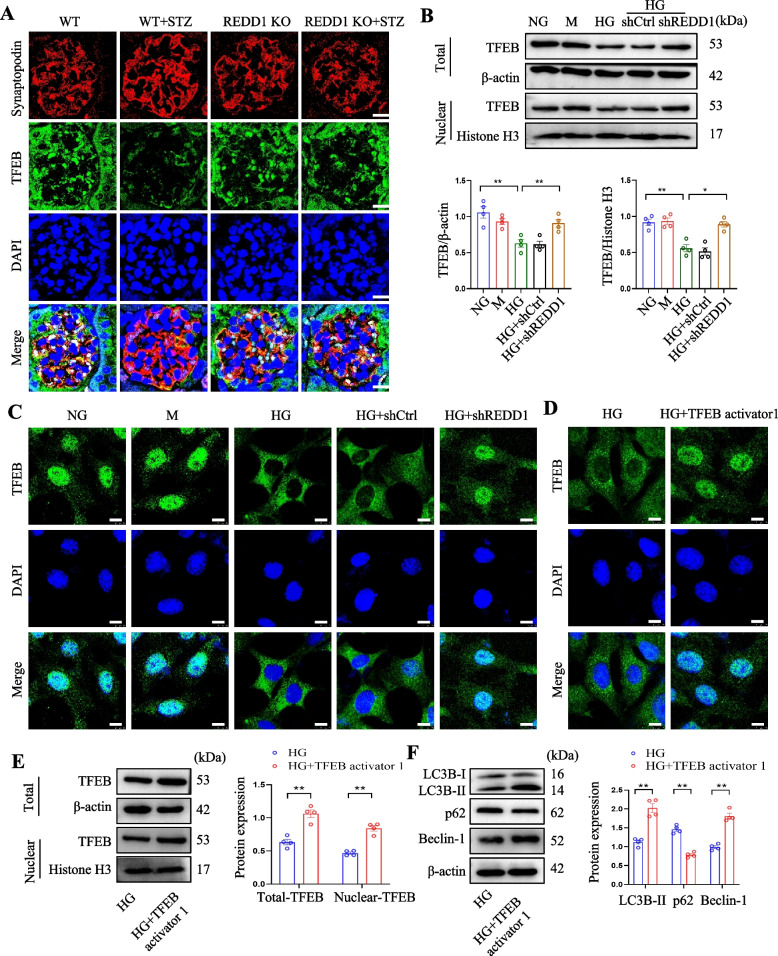
REDD1 deficiency promotes activation of TFEB in podocytes under diabetic conditions. **A** Representative immunofluorescence confocal images for TFEB, synaptopodin, and DAPI in kidney sections (scale bar, 20 μm). **B** Representative western blot images and quantification of TFEB in total and nuclear fractions of MPCs (*n* = 4). **C**,** D** Representative immunofluorescence images of TFEB in podocytes under the indicated conditions (scale bar, 7.5 μm). **E** Representative western blot images and quantification of TFEB in total and nuclear fractions of MPCs (*n* = 4). **F** Representative western blot images and quantification of LC3B, Beclin-1, and p62 in MPCs (*n* = 4). NG: 5.6 mM D-glucose; M: NG + mannitol (24.4 mM); HG: 30 mM D-glucose; shCtrl: shRNA control plasmid; shREDD1: REDD1 shRNA plasmid. Data are presented as mean ± SEM. *P < 0.05, **P < 0.01

TFEB, a master regulator of autophagic flux and lysosomal biogenesis, has been shown to promote podocyte autophagy under diabetic conditions (Zhao et al. 2018). Immunofluorescence double-staining revealed that TFEB expression was decreased in podocytes of diabetic mice, and this change was reversed by REDD1 KO (Fig. 8A). HG treatment inhibited TFEB expression in MPCs, which was reversed by REDD1 interference (Fig. 8B). In addition, TFEB nuclear translocation, which reflects TFEB activity, was significantly decreased in HG group compared to NG group, and this alteration was reversed by REDD1 knockdown (Fig. 8B). Similarly, immunofluorescence staining showed that HG decreased nuclear localization of TFEB in MPCs, which was restored by REDD1 silencing (Fig. 8C). Next, we evaluated the effect of TFEB activation on autophagy in MPCs exposed to HG. We found that TFEB activator 1 (compound C1) significantly increased TFEB expression and nuclear translocation in MPCs under HG conditions (Fig. 8D, E). Meanwhile, TFEB activator 1 upregulated levels of LC3B-II and Beclin-1 and reduced p62 expression in HG-treated MPCs, indicating TFEB activator 1 enhances podocyte autophagic activity (Fig. 8F). Taken together, these results suggest that REDD1 deficiency restores autophagic activity via promoting TFEB nuclear translocation in podocytes under diabetic conditions.

### REDD1 deficiency reduces SFN expression in podocytes under diabetic conditions

**Fig. 9:**
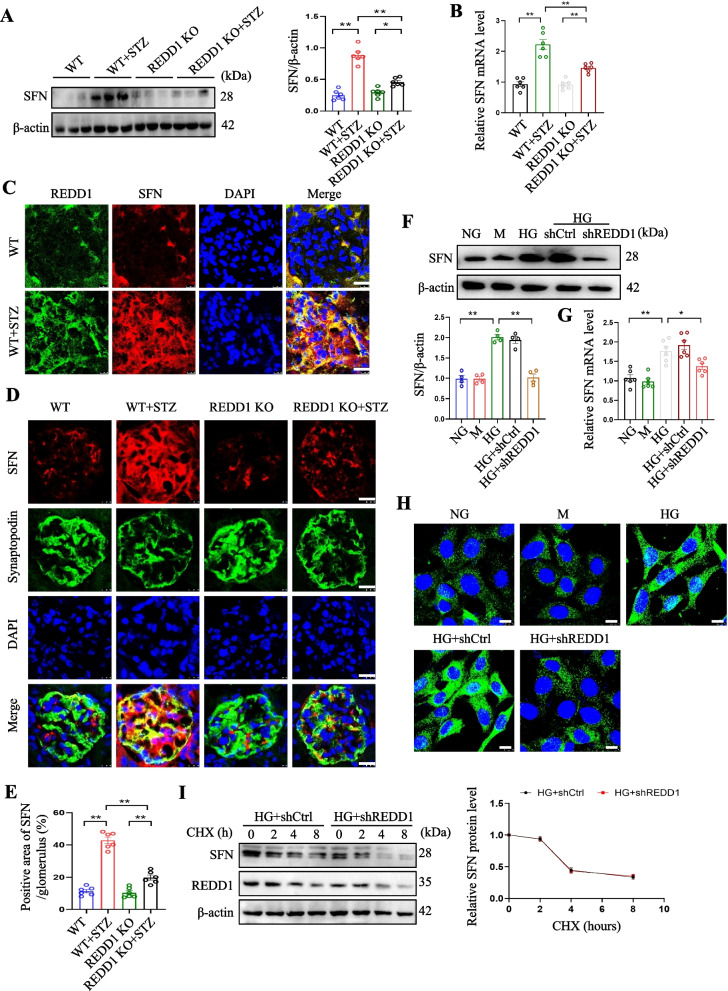
REDD1 deficiency alleviates SFN expression in podocytes under diabetic conditions. **A** Representative western blot images and quantification of SFN protein in renal cortical tissues (*n* = 6). **B** Quantitative analysis of SFN mRNA expression in renal cortical tissues (*n* = 6). **C** Representative double immunofluorescence images showing the co-localization of REDD1 and SFN in glomeruli (scale bar, 20 μm). **D** Representative immunofluorescence confocal images for SFN, synaptopodin, and DAPI in kidney sections (scale bar, 20 μm). **E** Quantitative analysis of SFN expression in glomeruli (*n* = 6). **F** Representative western blot images and quantitative analysis of SFN in MPCs (*n* = 4). **G** RT-qPCR analysis of SFN mRNA levels in MPCs (*n* = 6). **H** Representative immunofluorescence images of SFN in MPCs (scale bar, 7.5 μm). **I** Cycloheximide chase assays assessing the impact of REDD1 on SFN stability in MPCs. (*n* = 3). NG: 5.6 mM D-glucose; M: NG + mannitol (24.4 mM); HG: 30 mM D-glucose; shCtrl: shRNA control plasmid; shREDD1: REDD1 shRNA plasmid. Data are presented as mean ± SEM. *P < 0.05, **P < 0.01

Stratifin (SFN, also named 14–3-3σ), a member of the 14–3-3 protein family, is a highly conserved, multi-function soluble acidic protein (Medina et al. 2007). Emerging evidence has demonstrated that SFN is closely related to both cisplatin- and hypoxia/reperfusion (H/R)-induced acute kidney injury and unilateral ureteral obstruction (UUO)-induced renal fibrosis in mice (Wang et al. 2022; Wang et al. 2024). In addition, it has been reported that REDD1 regulates mTORC1 signaling by interacting with 14–3-3 proteins (DeYoung et al. 2008). Therefore, we evaluated the effect of REDD1 deficiency on SFN expression in podocytes under diabetic conditions. SFN expression was significantly elevated in the kidney tissues of diabetic mice compared with non-diabetic WT controls, and this increase was markedly attenuated by REDD1 KO (Fig. 9A). We also found that SFN mRNA level was significantly increased in the kidneys of diabetic mice, and this increase was alleviated by REDD1 knockout (Fig. 9B). Meanwhile, immunofluorescence staining revealed co-localization of REDD1 and SFN in the glomeruli (Fig. 9C). Immunofluorescence double staining showed that SFN expression was increased in podocytes of diabetic mice, and this increase was reduced by REDD1 deletion (Fig. 9D). Similarly, REDD1 deletion significantly reduced glomerular SFN expression in diabetic mice (Fig. 9D, E). Moreover, we found that HG stimulation induced SFN expression in MPCs, and this induction was suppressed by REDD1 knockdown (Fig. 9F). Furthermore, HG-induced mRNA expression of SFN in MPCs was markedly inhibited by REDD1 interference (Fig. 9G). Consistently, immunofluorescence staining further confirmed that HG stimulation upregulated SFN expression, which was also attenuated by REDD1 knockdown (Fig. 9H). Next, we assessed whether REDD1 influenced SFN protein stability using a CHX chase assay in MPCs. As shown in Fig. 9I, the half-life of SFN had no difference in HG-treated MPCs with shREDD1 or shCtrl transfection, suggesting that REDD1 knockdown does not affect SFN protein stability.

### REDD1 deficiency alleviates HG-induced PANoptosis via inhibiting SFN-RIPK3 signaling in podocytes

**Fig. 10:**
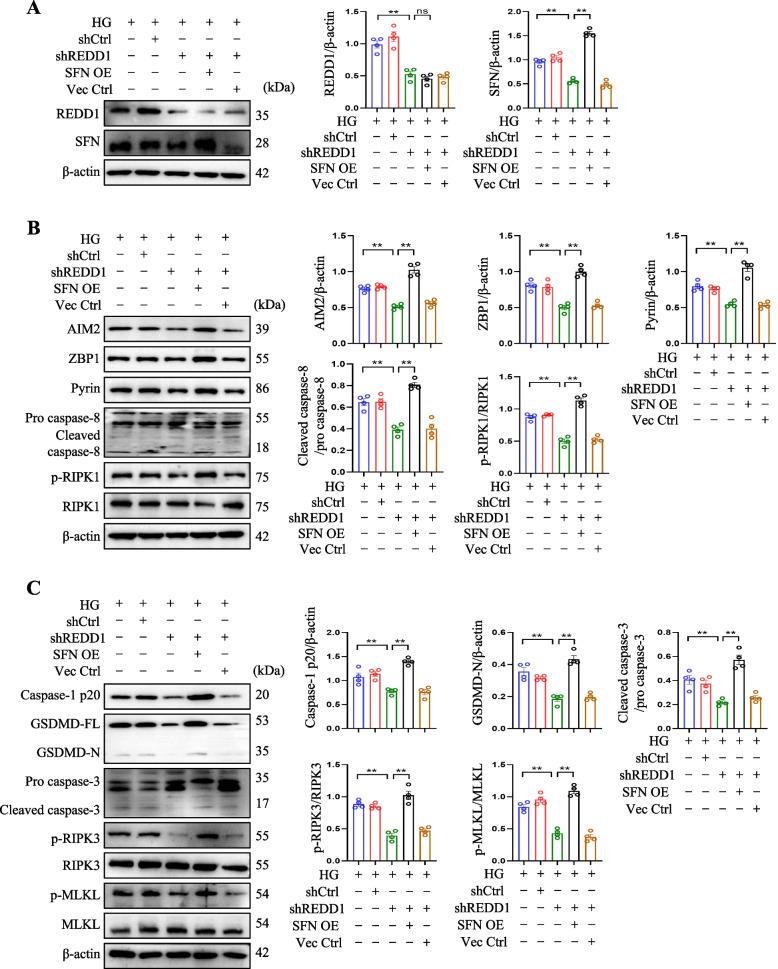
REDD1 silencing alleviates HG-induced PANoptosis via modulating SFN-RIPK3 signaling in podocytes. **A** Representative western blot images and quantification of REDD1 and SFN in MPCs. **B** Western blot analysis and quantification of PANoptosome components (AIM2, ZBP1, Pyrin, caspase-8, RIPK1, and p-RIPK1). **C** Representative western blot images and quantification of key PANoptosis-related proteins (caspase-1 p20, GSDMD-N, cleaved caspase-3, RIPK3, MLKL, and their phosphorylated forms) in MPCs. HG: 30 mM D-glucose; shCtrl: shRNA control plasmid; shREDD1: REDD1 shRNA plasmid; SFN OE: SFN overexpression; Vec ctrl: vector control; siNC: siRNA negative control; siSFN: SFN siRNA. Data are presented as mean ± SEM (*n* = 4). ns: no significance. **P < 0.01

**Fig. 11:**
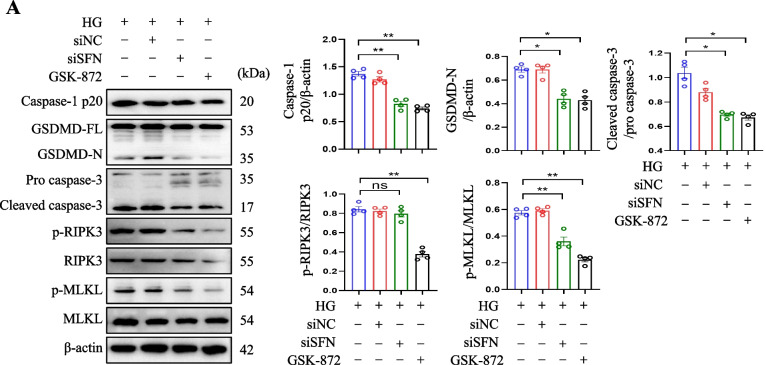
Inhibition of SFN or RIPK3 alleviates HG-induced PANoptosis in podocytes. **A** Representative western blot images and quantitative analysis of core PANoptosis-related protein expression. HG: 30 mM D-glucose; siNC: siRNA negative control; siSFN: SFN siRNA. Data are presented as mean ± SEM (*n* = 4). ns: no significance. *P < 0.05, **P < 0.01

Previous study confirmed that SFN bound directly to RIPK3, and SFN-RIPK3 mediated cisplatin- and H/R-induced renal tubular cell injury (Wang et al. 2022). To further elucidate the role of SFN and RIPK3 in REDD1 promoting PANoptosis in podocytes under diabetic conditions, MPCs were transfected with SFN expression plasmid and REDD1 shRNA. We found that SFN transfection significantly promoted SFN expression in HG-treated MPCs with REDD1 interference (Fig. 10A). SFN overexpression had no effect on REDD1 expression in MPCs exposed to HG (Fig. 10A). Next, we evaluated whether SFN overexpression influenced the protective effects of REDD1 deficiency on PANoptosis. We found that SFN overexpression counteracted the inhibitory effect of REDD1 knockdown on PANoptosome assembly, as evidenced by restored expression of AIM2, ZBP1, Pyrin, caspase-8, and p-RIPK1 (Fig. 10B). In addition, SFN overexpression abrogated the inhibitory effect of REDD1 knockdown on the upregulation of core PANoptosis-associated proteins, including caspase-1 p20, GSDMD-N, cleaved caspase-3, p-RIPK3 and p-MLKL (Fig. 10C). To investigate whether SFN and RIPK3 contribute to REDD1-mediated PANoptosis in podocytes under HG conditions, MPCs were transfected with SFN siRNA or treated with RIPK3 pharmacological inhibitor GSK-872. We found that HG-induced activation of key PANoptosis-related proteins was suppressed by SFN silencing or GSK-872 treatment (Fig. 11A). Collectively, these results indicate that REDD1 contributes to HG-induced podocyte PANoptosis via promoting SFN-RIPK3 signaling.

### REDD1 deficiency restores autophagy via SFN-RIPK3-TFEB axis in podocytes exposed to HG

**Fig. 12:**
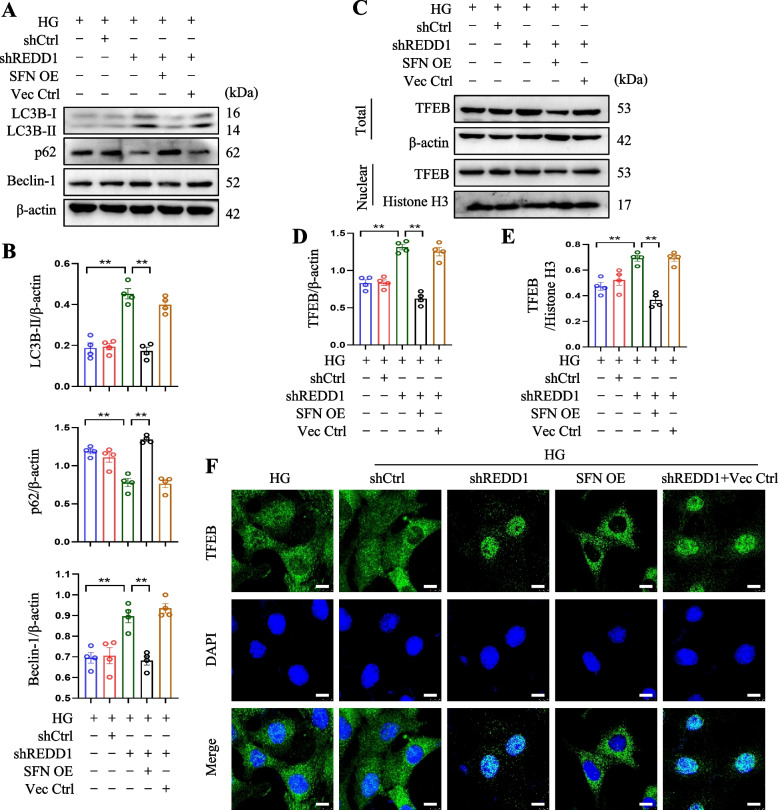
SFN overexpression retards the restoring of autophagy by REDD1 knockdown in podocytes exposed to HG. **A**, **B** Representative western blot images and quantification of LC3B, p62, and Beclin-1 in MPCs (*n* = 4). **C**-**E** Representative western blot images and quantification of total and nuclear TFEB levels in MPCs (*n* = 4). **F** Representative immunofluorescence images of TFEB in MPCs (scale bar, 7.5 μm). HG: 30 mM D-glucose; shCtrl: shRNA control plasmid; shREDD1: REDD1 shRNA plasmid; SFN OE: SFN overexpression; Vec ctrl: vector control. Data are presented as mean ± SEM. **P < 0.01

**Fig. 13:**
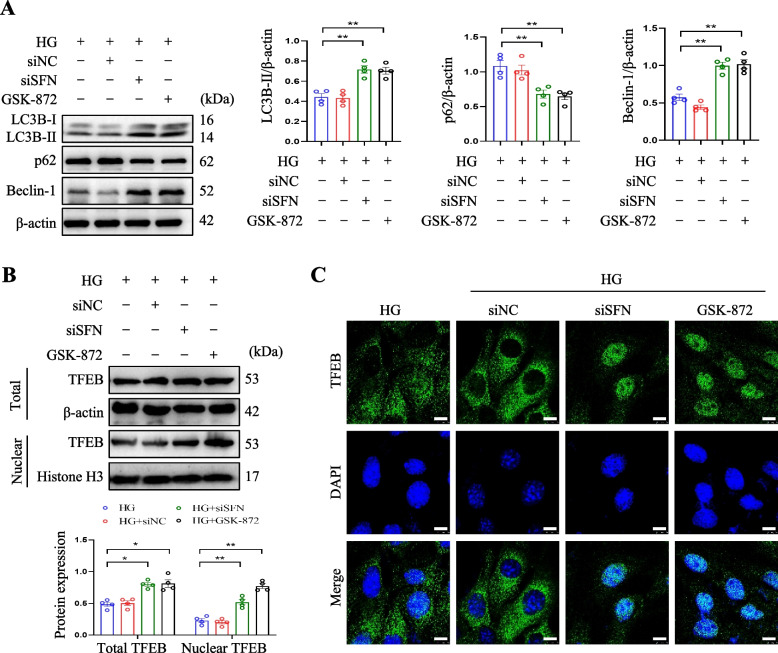
SFN knockdown or GSK-872 alleviates autophagic dysfunction in podocytes under HG conditions. **A** Representative western blot images and quantification of LC3B, p62, Beclin-1 in MPCs. **B** Representative western blot images and quantification of total and nuclear TFEB in MPCs. **C** Representative immunofluorescence images of TFEB in MPCs (scale bar, 7.5 μm). HG: 30 mM D-glucose; siNC: siRNA negative control; siSFN: SFN siRNA. Data are presented as mean ± SEM (*n* = 4). *P < 0.05, **P < 0.01

REDD1 knockdown increased LC3B-II and Beclin-1 expressions and reduced p62 expression in HG-treated MPCs, and these changes were reversed by SFN overexpression (Fig. 12A, B). In addition, the increased expression of TFEB and TFEB nuclear translocation induced by REDD1 knockdown was blunted by SFN overexpression in MPCs under HG conditions (Fig. 12C-E). Immunofluorescence analysis further revealed that the promotional effect of REDD1 interference on TFEB nuclear translocation was abrogated by SFN overexpression (Fig. 12F). Moreover, both SFN knockdown and RIPK3 inhibitor GSK-872 significantly promoted LC3B-II and Beclin-1 expressions and inhibited p62 expression in HG-treated MPCs (Fig. 13A). As expected, we also found that both SFN silence and GSK-872 significantly increased TFEB expression and nuclear translocation in MPCs exposed to HG (Fig. 13B, C).

**Fig. 14:**
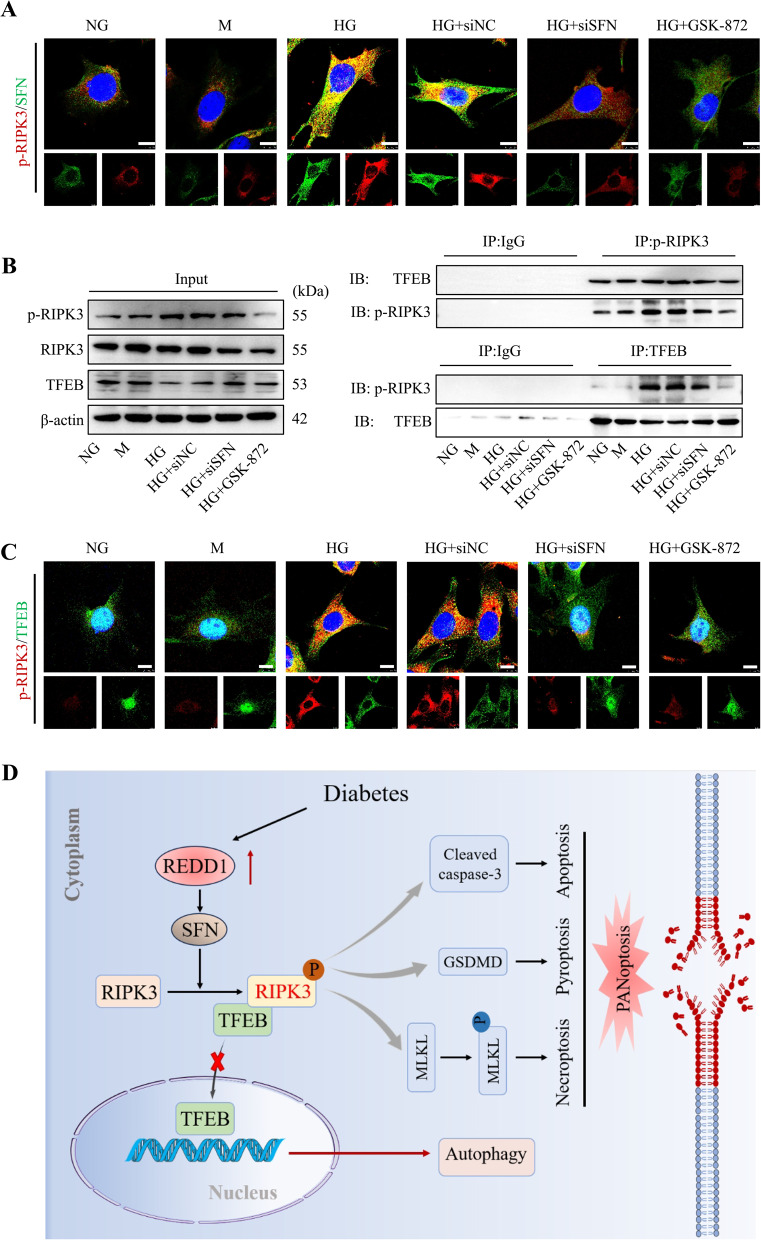
The interaction between SFN and p-RIPK3, and between p-RIPK3 and TFEB in MPCs*. ***A** Representative immunofluorescence images showing the co-localization of SFN and p-RIPK3 in MPCs (scale bar, 7.5 μm). **B** Co-IP assays demonstrating the direct interaction between p-RIPK3 and TFEB in MPCs. **C** Representative immunofluorescence images showing the co-localization of p-RIPK3 and TFEB, and the subcellular localization of p-RIPK3 and TFEB in MPCs (scale bar, 7.5 μm). **D** A schematic summary of REDD1-mediated podocyte PANoptosis and autophagy dysfunction via regulating SFN-RIPK3 pathway in DKD (This schematic diagram was created by using Power Point. Third-party graphical elements were adapted with modifications and alterations). NG: 5.6 mM D-glucose; M: NG + mannitol (24.4 mM); HG: 30 mM D-glucose; siNC: siRNA negative control; siSFN: SFN siRNA

To define the mechanism linking the SFN-RIPK3 axis to TFEB suppression, we confirmed their spatial association. Immunofluorescence revealed enhanced cytoplasmic co-localization of SFN with p-RIPK3 under HG conditions, which was diminished by SFN knockdown or GSK-872 treatment (Fig. 14A). Co-IP assays demonstrated a direct interaction between p-RIPK3 and TFEB in MPCs (Fig. 14B). This interaction was strengthened by HG and attenuated by SFN knockdown or GSK-872 treatment (Fig. 14B). In addition, HG increased the cytoplasmic co-localization of p-RIPK3 with TFEB and reduced nuclear TFEB expression, and these changes were reversed by SFN knockdown and GSK-872 treatment (Fig. 14C). Taken together, these data suggest that SFN-RIPK3-TFEB axis mediates REDD1-driven autophagy impairment in podocytes under HG conditions.

## Discussion

Podocyte injury is a central event in the pathogenesis of glomerular diseases such as focal segmental glomerulosclerosis, glomerulonephritis, genetic forms of nephrotic syndrome and DKD (Torban et al. 2019). In this study, we observed significant upregulation of REDD1 in kidney tissues in STZ-induced diabetic mice and HG-treated MPCs. In a mouse model of STZ-induced diabetes, genetic deletion of REDD1 alleviated renal functional decline, structural abnormalities, and podocyte loss, suggesting that REDD1 is a key mediator of podocyte injury under diabetic conditions. Notably, we provide the first evidence that REDD1 deficiency significantly attenuates podocyte PANoptosis in diabetic mice. Furthermore, in vitro experiments demonstrated that REDD1 silencing reduces HG-induced PANoptosis and restores HG-impaired autophagy by regulating the SFN/RIPK3 pathway.

As essential components of the filtration barrier, podocytes contribute to the maintenance of basement membrane integrity (Brinkkoetter et al. 2013). Consequently, podocyte injury leads to proteinuria, an early clinical manifestation of glomerular disease. Morphometric analyses of renal biopsies from diabetic patients have shown that reduced podocyte density precedes microalbuminuria and serves as a strong predictor of disease progression (Shankland 2006). However, the underlying mechanisms of podocyte injury in DKD remain incompletely understood. In our present study, we found that REDD1 deletion prevented proteinuria, podocyte loss, and renal injury in STZ-induced diabetic mice, and these findings are consistent with previous reports (Sunilkumar et al. 2022). We also found that REDD1 silencing mitigated cytoskeletal disorganization, loss of mitochondrial membrane potential, and mitochondrial structural damage in MPCs exposed to HG. These findings suggest that REDD1 upregulation represents an early mechanism contributing to podocyte injury and subsequent proteinuria in DKD.

PANoptosis is a coordinated inflammatory cell death pathway integrating key features of pyroptosis, apoptosis, and necroptosis, and has been implicated in various systemic diseases including infectious diseases, cancer, neurodegenerative disorders, and inflammatory diseases (Zhu et al. 2023). Recent studies have indicated that PANoptosis serves an important role in various kidney injury models, including those induced by ischemia/reperfusion (I/R), unilateral ureteral obstruction (UUO) and exposure to nephrotoxic agents, such as cisplatin, citrinin, and aristolochic acid (Zhuang et al. 2025; Zhang et al. 2025; Lin et al. 2024; Wang et al. 2025b; Xu et al. 2024). In addition, podocyte PANoptosis in lupus nephritis and DKD has been reported (Wang et al. 2025a; Lv et al. 2025). However, its role in DKD remains largely unexplored. Here, we found that podocyte PANoptosis was increased in diabetic mice and HG-treated MPCs, unveiling a novel mechanism of renal injury in diabetes. Moreover, we demonstrated that REDD1 deletion alleviated podocyte PANoptosis in diabetic mice. And the increased podocyte PANoptosis induced by HG was retarded by REDD1 silencing. Therefore, our data suggest that REDD1 deficiency improves diabetic kidney injury by inhibiting PANoptosis of podocytes.

In this study, we identified SFN as a critical downstream effector of REDD1. SFN is a highly conserved, soluble acidic protein within the 14–3-3 family that serves crucial roles in diverse cellular processes, including proliferation, differentiation, death, and inflammation (Li et al. 2024; Sun et al. 2015; Sime et al. 2018; Munier et al. 2021). It has been reported that REDD1 modulates TSC1/2-mTOR signaling via 14–3-3 shuttling (DeYoung et al. 2008). The 14–3-3 protein family, especially the 14–3-3σ protein, is upregulated in several kidney injury models (UUO, I/R, and nephrotoxic serum administration) and in biopsies from IgA nephropathy and membranous nephropathy patients (Rizou et al. 2018). In addition, SFN knockdown impeded UUO-induced kidney injury and interstitial fibrosis in mice (Wang et al. 2024). Moreover, in a mouse model of cisplatin-induced AKI, SFN knockdown improved renal function and kidney injury, reduced necroptosis, and the inflammatory response (Wang et al. 2022). In our study, we observed increased expression of protein and mRNA of SFN in kidney tissues of STZ-induced diabetic mice, which was retarded by REDD1 KO. Meanwhile, HG-induced SFN expression was suppressed by REDD1 interference in MPCs. Notably, we also found SFN knockdown prevented HG-induced PANoptosis in MPCs. Interestingly, the inhibitory effect of REDD1 knockdown on podocyte PANoptosis was reversed by SFN overexpression. Previous study demonstrated that SFN mediates necroptosis by directly binding to RIPK3 in cisplatin-or H/R-treated tubular epithelial cells (Wang et al. 2022). Importantly, the enhanced cytoplasmic co-localization of SFN with p-RIPK3 under HG conditions suggests a functional interaction. This interaction was effectively disrupted by either SFN knockdown or pharmacological inhibition of RIPK3, indicating that the SFN-p-RIPK3 axis is a critical and targetable node in hyperglycemia-induced podocyte injury. A recent study has reported that RIPK3 forms homo-aggregates to sequentially recruit MLKL, RIPK1, and caspase-8, concurrently triggering necroptosis, apoptosis, and pyroptosis within the same cell to drive PANoptosis (Yang et al. 2026). In our study, we showed that RIPK3 inhibitor GSK-872 inhibited HG-induced activation of key PANoptosis-related proteins in MPCs. Taken together, these data indicate that REDD1 may modulate PANoptosis by regulating the SFN-RIPK3 pathway in MPCs.

Autophagy serves as an essential catabolic process, and its dysregulation has been implicated in DKD through multiple signaling pathways (Stanigut et al. 2025). REDD1 has been reported to promote or inhibit autophagy in a context-dependent manner (Chen et al. 2022; Qiao et al. 2015). In this study, we observed elevated REDD1 expression and impaired autophagic flux in podocytes of diabetic mice, and REDD1 deletion restored autophagy. Consistently, HG-induced autophagy impairment was reversed by REDD1 knockdown in vitro. In addition, we also found that REDD1 knockdown effectively restored autophagic flux by enhancing autophagosome biogenesis. These data indicate that REDD1 deficiency protects against podocyte damage by ameliorating autophagy impairment in DKD.

TFEB is an important transcription factor recognized as the master regulator of autophagy and lysosomal biogenesis (Settembre et al. 2011). Our recent study showed that TXNIP deficiency inhibited HG-induced tubular epithelial cell injury by promoting TFEB-mediated autophagy (Du et al. 2024). Studies have reported that enhancing TFEB-mediated autophagy in podocytes may represent an important therapeutic strategy for DKD (Zhao et al. 2018; Hou et al. 2020). In this study, we found that SFN knockdown or GSK-872 promoted TFEB expression and nuclear translocation, and reversed autophagy dysfunction in podocytes exposed to HG. Meanwhile, SFN overexpression alleviated the promotional effect of REDD1 interference on autophagy and TFEB nuclear translocation. Recent evidence suggests that RIPK3 interacts with TFEB, inhibiting its nuclear translocation and impairing autophagic flux in septic AKI mice and LPS-treated cultured tubular epithelial cell (Li et al. 2021). In this study, Co-IP and immunofluorescence data confirmed the potential interaction of RIPK3 with TFEB in podocytes. Taken together, these data suggest that REDD1 deficiency restores podocyte autophagy via regulating SFN-RIPK3-TFEB signaling in DKD. Previous studies demonstrated that RIPK3 contains an N-terminal kinase domain and a C-terminal RHIM motif (Meylan and Tschopp 2005; Sun et al. 2012), and its phosphorylation is essential for its kinase activity (Frank and Vince 2019). TFEB subcellular distribution is governed by phosphorylation via multiple serine/threonine kinases including mTOR, ERK, and calcineurin (Napolitano and Ballabio 2016; Puertollano et al. 2018). In our study, we showed that GSK-872 inhibited RIPK3 phosphorylation and promoted TFEB nuclear translocation in podocytes exposed to HG. However, whether RIPK3 regulate TFEB via direct phosphorylation in podocyte need to be investigated further.

## Conclusion

In conclusion, our findings demonstrate that REDD1 plays an important role in podocyte PANoptosis and autophagy dysfunction via regulating the SFN-RIPK3 pathway in DKD (Fig. 14D). These findings implicate that inhibition of REDD1 could be a potential therapeutic target for DKD.

## Data Availability

No datasets were generated or analysed during the current study.
